# Evidence of unidirectional hybridization and second‐generation adult hybrid between the two largest animals on Earth, the fin and blue whales

**DOI:** 10.1111/eva.13091

**Published:** 2020-08-28

**Authors:** Christophe Pampoulie, Davíð Gíslason, Guðbjörg Ólafsdóttir, Valérie Chosson, Sverrir Daníel Halldórsson, Stefano Mariani, Bjarki Þ. Elvarsson, Marianne H. Rasmussen, Maria R. Iversen, Anna Kristín Daníelsdóttir, Gísli A. Víkingsson

**Affiliations:** ^1^ Marine and Freshwater Research Institute Hafnarfjörður Iceland; ^2^ Matís ohf. Reykjavík Iceland; ^3^ School of Biological and Environmental Sciences Liverpool John Moores University Liverpool UK; ^4^ The University of Iceland’s Research Center in Húsavík Húsavík Iceland

**Keywords:** *Balaenoptera* sp., directionality, F2 hybrid, hybridization, marine mammals

## Abstract

Biodiversity in the oceans has dramatically declined since the beginning of the industrial era, with accelerated loss of marine biodiversity impairing the ocean's capacity to maintain vital ecosystem services. A few organisms epitomize the damaging and long‐lasting effects of anthropogenic exploitation: Some whale species, for instance, were brought to the brink of extinction, with their population sizes reduced to such low levels that may have caused a significant disruption to their reproductive dynamics and facilitated hybridization events. The incidence of hybridization is nevertheless believed to be rare, and very little information exists on its directionality. Here, using genetic markers, we show that all but one whale hybrid sample collected in Icelandic waters originated from the successful mating of male fin whale and female blue whale, thus suggesting unidirectional hybridization. We also demonstrate for the first time the existence of a second‐generation adult (male) hybrid resulting from a backcross between a female hybrid and a pure male fin whale. The incidence of hybridization events between fin and blue whales is likely underestimated and the observed unidirectional hybridization (for F1 and F2 hybrids) is likely to induce a reproductive loss in blue whale, which may represent an additional challenge to its recovery in the Atlantic Ocean compared to other rorquals.

## 1 INTRODUCTION

From the 12th to the 20th century, the hunt of whales throughout their distribution range led to the near extinction of several species (Gambell, 1993; McVay, 1966), which greatly impacted most of marine ecosystems (Clapham & Link, 2006; Croll, Kudela, & Tershy, 2006) and then compelled drastic changes to conservation strategies. Many species of closely related cetaceans were reduced to population sizes that may have facilitated hybridization due to the rarity of conspecifics, hence increasing the risk of genetic swamping and possibly threatening the persistence of species.

Hybridization among closely related species is an important evolutionary phenomenon (Mallet, 2007) and has been reported in several animal taxa (Schwenk, Brede, & Streit, 2008). Large marine mammals such as fin whale (*Balaenoptera physalus*) and blue whale (*B. musculus*) are no exception, with alleged hybrids reported as early as 1887 during commercial whaling operations along the Lapland coast, when Cocks (Cocks, 1887) mentioned the presence of so‐called “bastards” among the fin and blue whalers. Another alleged hybrid was reported almost 80 years later off Kodiak Island in 1965 (Doroshenko, 1970). Twenty years later, hybridization between fin and blue whales was eventually demonstrated for the first time using genetic tools (Árnason, Spilliaert, Pálsdóttir, & Árnason, 1991; Bérubé & Aguilar, 1998; Spilliaert et al., 1991): A whale caught in 1983 in Icelandic waters was classified as a hybrid between a fin whale mother and a blue whale father, while two whales caught in 1986 and 1989 were found to be hybrids between a fin whale father and a blue whale mother (Árnason et al., 1991; Spilliaert et al., 1991). A further case was documented in the northwest of Spain in 1984, where a female rorqual was genetically identified as a hybrid between a fin whale father and a blue whale mother (Bérubé & Aguilar, 1998). Baleen whales are elusive and relatively difficult to study, which likely affects reporting of hybrids and introgressive events. Scientists often rely on expensive biopsy sampling cruises, stranded animals, or aboriginal/commercial whaling operation to obtain genetic samples from large marine mammals. While current commercial whaling activities remain a highly controversial issue in conservation fora, they also provide unique and opportunistic access to sample and therefore to crucial biodiversity information. In recent years, commercial whaling operations in Iceland have led to the discovery of three more alleged hybrids, one in 2013 and two in 2018, while one living hybrid is known to regularly visit the Skjálfandi Bay (Northeast Iceland) almost every year since 2012. Here we screen the largest collection of hybrids between the two largest mammals on Earth using 24 microsatellite and one mitochondrial DNA marker (mtDNA), with the aim to verify directionality of hybridization and explore the potential for hybrid fertility.

## 2 MATERIALS AND METHODS

### 2.1 Sampling

**Figure 1:**
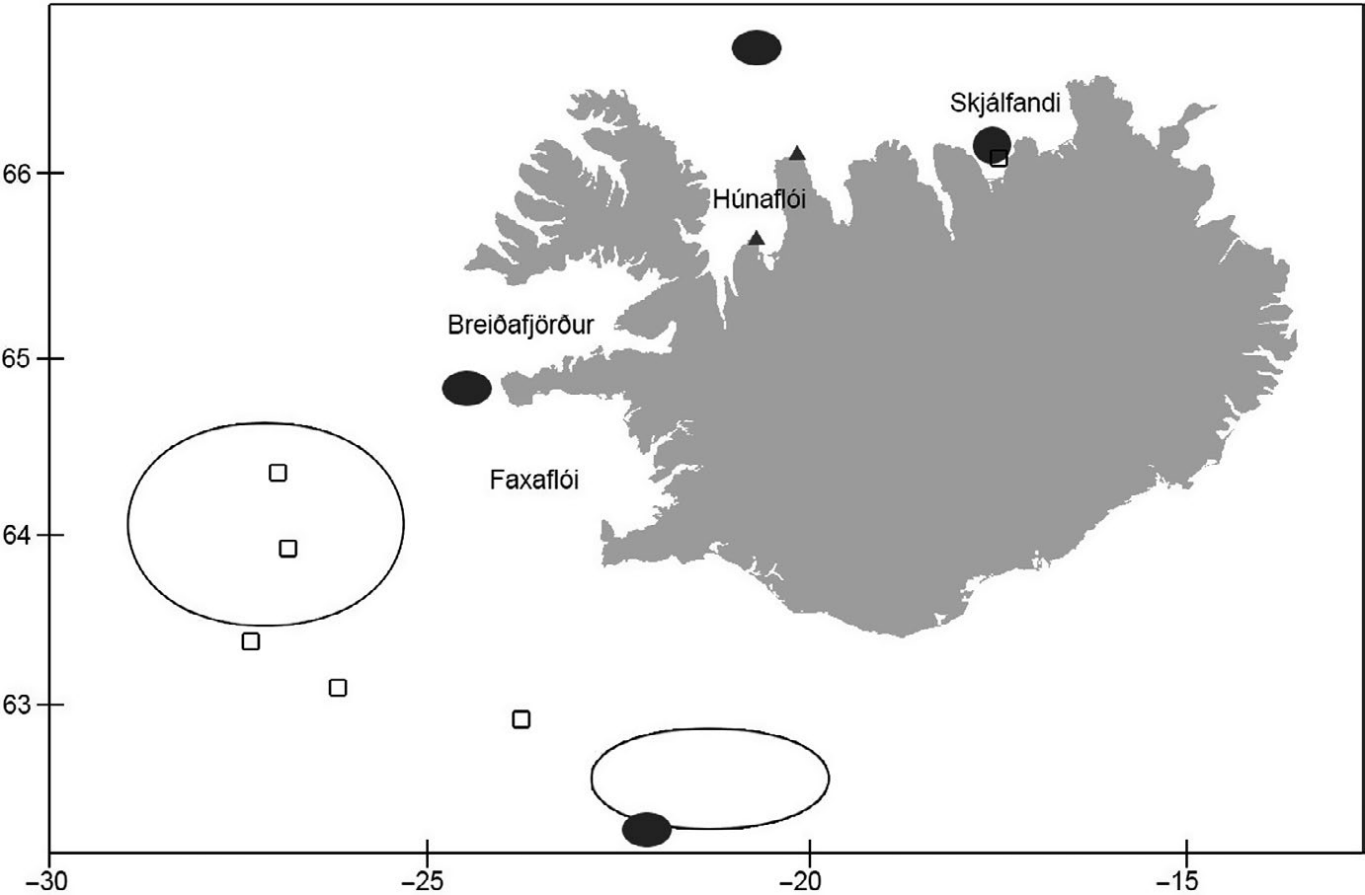
Map of sample area of fin (empty circles) and blue (gray area) whales. Hybrid location is denoted with square. The two gray triangles on land depict stranded blue whales

Fin whale genetic samples were routinely collected during the commercial whaling operation in 2018; 34 samples were randomly selected among the ones exhibiting standard pure phenotypic features of fin whale. The hybrid samples of 2013 and 2018 were also collected during commercial whaling operations in Iceland, while those of 1986 and 1989 were collected during a scientific research project according to a special permit (see https://iwc.int/table_permit). Blue whale samples come from stranded (*N* = 2) and biopsied (*N* = 25) individuals. All samples were collected in Icelandic waters (Figure 1). For both species, samples were collected <24 hr after stranding, biopsy or death of the animal, put in 96% ethanol and conserved at 5°C until analyses in the laboratory. Information on individual sex and date of capture is presented in supplementary materials when available (Table S1).

The six suspected hybrid samples analyzed during this study (Table 1) include the confirmed hybrids caught in 1986 and 1989, the suspected living hybrid from Skjálfandi Bay, and the hybrids caught in 2013 (*n* = 1) and 2018 (*n* = 2). No tissues from the 1983 hybrid or from the fetus found in the 1986 hybrid could be retrieved due to sample loss.

**Table 1:** Characteristics of the fin × blue hybrids genetically analyzed to date

Name	Size (m)	Sex	Age	Maturity	mtDNA sequence	Mother	Father	Generation
H1983^1^	19.81	♂	7	Immature	Fin whale	Fin whale	Blue whale	F1
**H1986** ^2^	21.33	♀	7	Pregnant with fetus	Blue whale	Blue whale	Fin whale	F1
**H1989** ^3^	21.03	♂	24	Immature	Blue whale	Blue whale	Fin whale	F1
**HALIVE**	na	♂	na	Courtship observed^a^	Blue whale	Blue whale	Fin whale	F1
**H2013**	20.70	♀	na	Mature	Blue whale	Blue whale	Fin whale	F1
**H2018−1**	21.33	♂	na	na	Blue whale	Blue whale	Fin whale	F1
**H2018−2**	18.23	♂	>20	na	Blue whale	F1 hybrid	Fin whale	F2
Spain 1984^4^	19.40	♀	4	Immature^b^	Blue whale	Blue whale	Fin whale	F1

Note

Size, sex, and age are depicted when available. The mtDNA sequences possible origin as well as interpretation of genetic data in terms of parental origin are mentioned. The hybrids analyzed during the present study are highlighted in bold. Na depicts nonavailable information.

^a^ Maria R. Iversen, personal communication. All biological information of hybrids caught in Icelandic waters was retrieved from the whale database of the Marine and Freshwater Research Institute of Iceland. Genetic information was retrieved from: ^1^Árnason et al. (1991); ^2^Árnason et al. (1991) and Spilliaert et al. (1991); ^3^Árnason et al. (1991); and ^4^Bérubé and Aguilar (1998), respectively. ^4^Biological information retrieved from Bérubé and Aguilar (1998).

^b^ Based on size comparison with fin and blue whales.

### 2.2 Microsatellite loci genotyping

A total of 15 microsatellite markers (Bérubé, Jorgensen, McEwing, & Palsbøll, 2000; Palsbøll, Bérubé, Larsen, & Jorgensen, 1997) and one sex marker (Bérubé & Palsboll, 1996) (ZFYX0582) were used in 3 multiplex polymerase chain reactions (PCRs). Multiplex 1 contained GATA098, EV1, ZFYX0582, GT310, EV37, and GT023. Annealing temperature was 54°C, and 32 PCR cycles were run. Multiplex 2 contained GATA417, GATA028, GT211, and GT575. Annealing temperature was 56°C, and 35 PCR cycles were run. Multiplex 3 contained GT195, GATA053, TAA023, GGAA520, GT271, and GT011. Annealing temperature was 58°C, and 32 PCR cycles were run.

PCRs were performed in a total volume of 10 μl consisting of 2 μl of DNA template (5–20 ng/µl), 0.1 µl Taq DNA polymerase, 1.0 μl of 10× Standard buffer, 0.8 μl dNTP (10 mM), 0.03–0.25 μl reverse and forward primers (100 μM), and dH_2_O up to final volume. The PCR was as follows: a 4‐min denaturation at 94°C followed by 32–35 cycles of 94°C denaturing for 30s, 54–58°C annealing for 50s and 68°C extension for 50s, plus a final extension of 7 min at 68°C. Amplified DNA fragments were separated by an ABI 3730 DNA Analyzer and were sized according to the GeneScan™‐500LIZ™ Size Standard. Alleles were scored manually with the GeneMapper™ Analysis Software version 4.1.

An additional set of nine microsatellite markers was used to offer greater assignment power of the hybrids to both parental species, namely GT541, GT129, GT227, AC082, TGAA610, CAAA074, CA232, GT122, and AC045 (Bérubé et al., 2005). Each marker was amplified by single PCR consisting of 2 μl of DNA template (5–20 ng/µl), 0,1 µl Taq DNA polymerase, 1 μl of 10× Standard buffer (New England BioLabs), 0.8 μl dNTP (10 mM), 0.1 μl reverse and forward primers (100 μM), and dH_2_O up to final volume. The PCR was identical to the one mentioned above but annealing temperatures varied from 55 to 63°C. The PCR products were combined in 3 multiplexes before loading it to the ABI 3730. Multiplex 4 contained GT541, GT227, CAAA074, and AC082. Multiplex 5 contained GT129, GT122, and AC045. Multiplex 6 contained TGAA610 and CA232.

### 2.3 Mitochondrial DNA D‐loop region genotyping

Since mitochondrial DNA (mtDNA) is inherited from the mother, we used a 285 bp fragment of the D‐loop region to assess the parental origin of the hybrids. Primers M.whale‐PCR‐F‐b (5′‐GATCGGTGGCCAACCCGTAGAAC‐3′) and MW‐PCR‐r (5′ GGTCCTGAAGTAAGAACCAGATG 3′) were designed at Matís Ltd. for the genetic identification of marine mammals and used to amplify the D‐loop region of all individuals. PCR was performed in a total volume of 20 μl composed of 2 μl of DNA template (5–20 ng/µl), 0.2 µl Taq DNA polymerase, 2.0 μl of 10 × Standard buffer, 0.4 μl dNTP (10 mM), 0.1 μl of each reverse and forward primers (100 μM), and 15.3 μl dH_2_O. PCR consisted of a 4 min denaturation at 94°C followed by 35 cycles of 94°C denaturing for 45s, 56°C annealing for 45s and 68°C extension for 1 min, plus a final extension of 7 min extension at 68°C. PCR fragment was then purified, and the purified PCR product was sequenced with forward or reverse using the following primers: M.whale‐Seq‐F‐B 5′‐CCAGTAGCTAGTCTTATCGAG‐3′, and MW_seq‐R_b1 5′‐TGGGCCCGGTGCGAGAAG‐3′. The PCR was performed as follows: 5 μl purified PCR product, 0.5 μl Big Dye, 1.5 μl of 5× buffer, 1 μl of either reverse or forward primers (3.5 μM), and 2.0 μl dH_2_O. The PCR consisted of 25 cycles of 96°C denaturing for 30s, 50°C annealing for 15 s and 60°C extension for 4 min. Sequencing was performed in ABI 3730 DNA Analyzer (Applied Biosystems). The software Sequencher v5.2.4 was used to align the forward and the reverse sequences for each sample and the consensus sequence exported.

### 2.4 Data analysis

Genetic diversity indices of the 24 microsatellite loci including the number of alleles (*n*), allelic size range (ASR), observed (*H*_O_) and expected (*H*_E_) heterozygosities, and departure from Hardy–Weinberg Equilibrium (HWE) within each samples for each locus were calculated in GENEPOP’007 (Rousset, 2008). Statistical significance for HWE was assessed using exact P‐values by Markov chain methods implemented in the same software. A principal component analysis (PCA) was performed using GenAlEX (Peakall & Smouse, 2006, 2012) to visualize relative multilocus genetic differences among parental species and hybrids.

Bayesian cluster analysis was performed using the program STRUCTURE (Pritchard, Stephens, & Donnelly, 2000) to assess the genetic relationship of the alleged hybrids to their potential parental species and was used to generate assignment probability of hybrids to both parental species. STRUCTURE investigates relationships among individuals of potential mixed and admixed origin. The program was run using an admixture model with correlated allele frequencies for *K* = 2 (representing the two separate whale species), for five iterations, each with a burn‐in period of 500,000 and MCMC replicates of 1,000,000. No prior information regarding species identification was considered.

The posterior distribution analysis of hybrid individuals falling into different categories was performed using the software NEWHYBRIDS (Anderson & Thompson, 2002), designed to support hybrids identification and backcross categories among two potential parental species. NEWHYBRIDS calculates the posterior probabilities of the hybrids to distinct hybrid types, namely F1 hybrid or backcross to either fin or blue whale. The program was run using default parameters over 15,000 burn‐in and 10,000 runs after burn‐in.

The final aligned set of mitochondrial DNA sequences contained 285 nucleotides of the mtDNA control region. The genealogy of the mtDNA sequences was assessed using a Maximum Parsimony tree implemented in MEGA‐X (Kumar, Stecher, Li, Knyaz, & Tamura, 2018). The analysis of mtDNA sequences was not performed to infer phylogeny but merely to verify the matrilineage of the hybrids, and hence infer directionality of hybridization.

## 3 RESULTS

Microsatellite loci diversity was usually higher in fin whale than blue whale, and this was reflected both at the mean number of alleles and at the species/loci heterozygosities level (Table 2). A total of 2 microsatellite loci were fixed for the fin whale (CAAA074, TGAA610; Table 2), while 4 were fixed for the blue whale (TGAA610, GT227, TAA023, GATA053; Table 2). Interestingly, some of these fixed microsatellite loci displayed alternative allele in both species, for example, TGAA610‐allele 143, GATA053‐allele 256, and GT227‐allele 122 were only found in blue whale (Table 2). In addition, several microsatellite loci displayed different allelic size range for both species, which make these loci powerful diagnostic markers for the identification of the hybrids (Table 2).

**Table 2:** Genetic diversity of the 24 microsatellite loci. Number of alleles (n) and allelic size range (ASR) at each locus, expected (*H*_e_) and observed (*H*_o_) heterozygosity are depicted for fin and blue whales

Locus	Fin whale				Blue whale			
	*n*	*ASR*	*H*_e_	*H*_o_	*n*	*ASR*	*H*_e_	*H*_o_
EV1	10	155–175	0.827	0.971	11	135–169	0.824	0.852
EV37	10	181–213	0.833	0.794	3	183–189	0.150	0.080
GT011	7	117–131	0.828	0.794	6	117–141	0.749	0.846
GT023	12	107–143	0.818	0.824	6	119–129	0.782	0.778
GT195	9	161–181	0.796	0.765	2	155–157	0.494	0.577
GT211	9	106–126	0.824	0.941	4	92–98	0.612	0.769
GT271	9	108–128	**0.728**	**0.706**	4	108–122	0.553	0.650
GT310	7	110–126	0.781	0.735	4	118–124	0.590	0.667
GT575	7	146–160	0.718	0.853	5	160–170	0.592	0.593
GATA028	12	191–235	0.891	0.853	6	171–191	0.680	0.741
GATA053	7	246–270	0.739	0.735	1	256	0.000	0.000
GATA098	7	96–120	0.780	0.824	7	88–128	0.778	0.815
GATA417	14	213–289	0.878	0.765	8	213–241	**0.850**	**0.704**
GGAA520	13	201–231	0.869	0.912	2	187–189	0.036	0.037
TAA023	6	86–104	0.639	0.765	1	86	0.000	0.000
AC082	5	128–140	0.167	0.177	6	132–158	0.600*	0.440*
CAAA074	1	148	0.000	0.000	3	144–152	0.518	0.482
GT227	3	126–130	0.058	0.059	1	122	0.000	0.000
GT541	6	82–104	0.752	0.794	7	90–106	0.687	0.741
AC045	8	194–208	0.790	0.735	3	188–192	0.575	0.482
GT122	14	155–187	0.833	0.882	2	137–139	0.384	0.444
GT129	5	88–106	0.585	0.559	2	94–96	0.105	0.111
CA232	5	144–172	0.526	0.471	8	126–178	0.717	0.704
TGAA610	1	147	0.000	0.000	1	143	0.000	0.000
**Overall loci**	7.79		0.652	0.662	4.38		0.472	0.482

Note

Values in bold indicate significant deviations from HWE (exact tests, *p* < .05).

**Figure 2:**
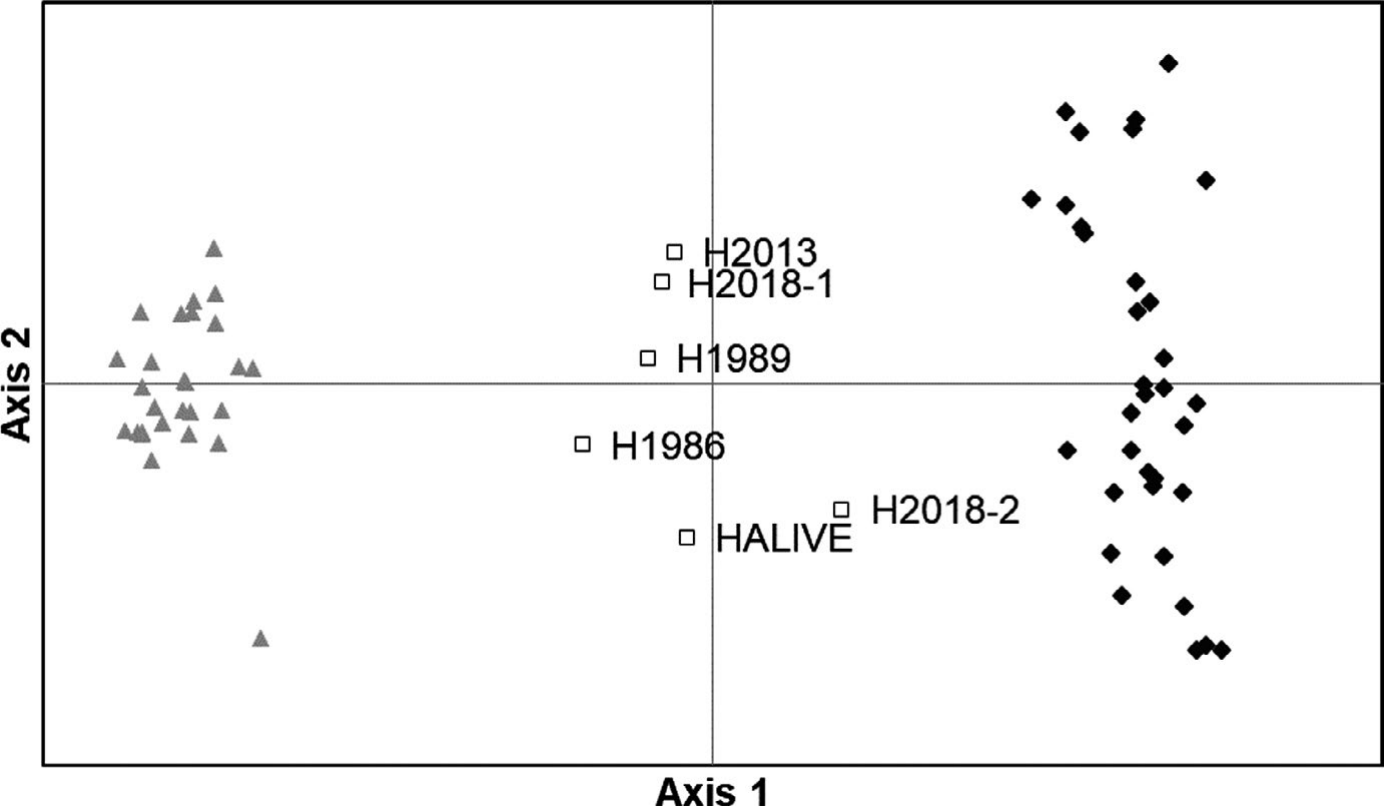
Principal component analyses (PCA) of the 24 microsatellite loci. Gray triangles indicate blue whale, filled diamond fin whale, and square hybrids. The first axis explained 37.5% of the variation and the second axis 2.8%

The PCA clearly separated the two investigated species, the fin and blue whale (Figure 2) with 37.5% of the variation explained by the first axis and 2.8% on the second axis. As expected, the hybrids were in the middle of the ordination, half‐way between fin and blue whale data points. The greater spread of fin whale specimens reflects their greater intraspecific variation compared to blue whale.

**Figure 3:**
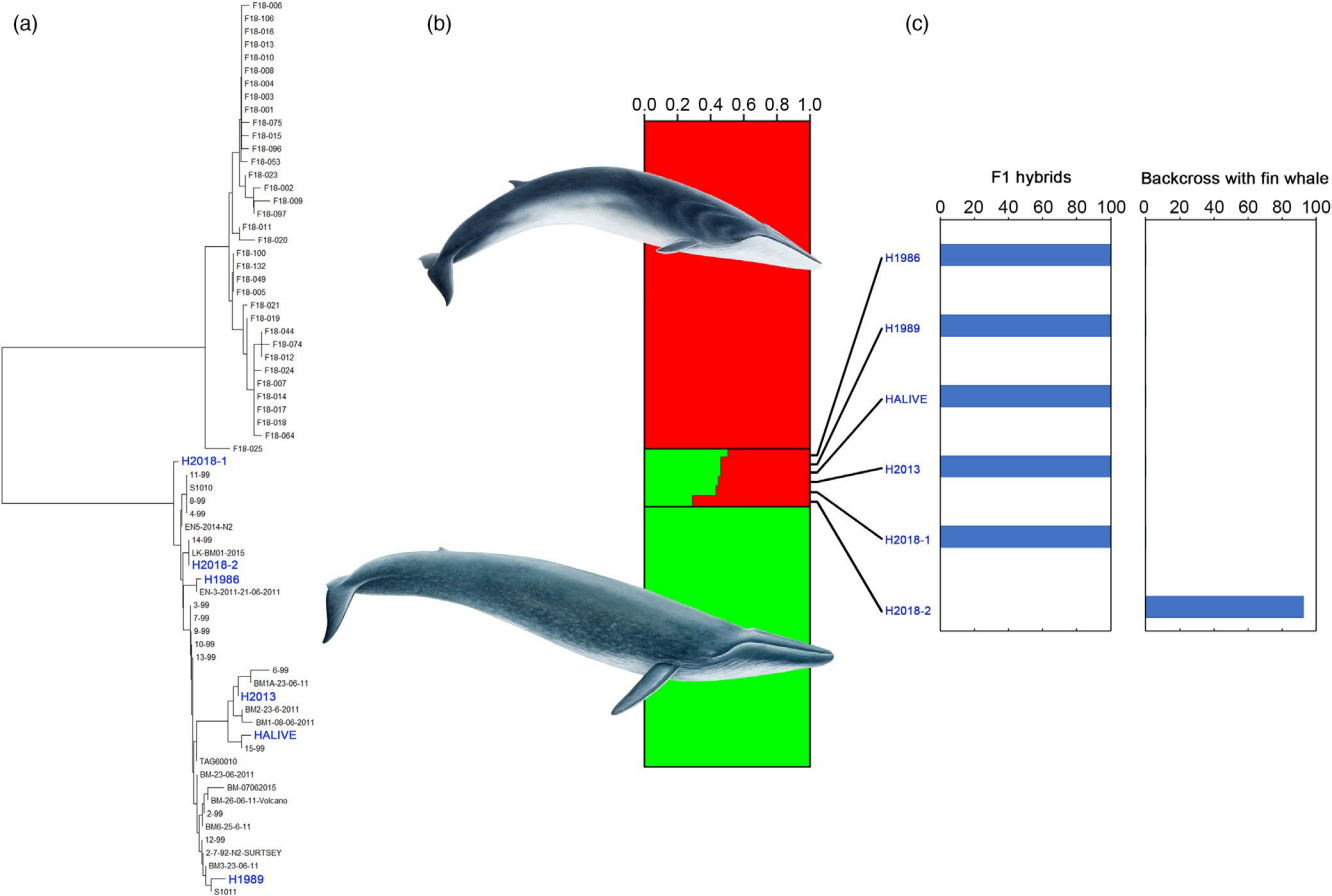
Genetic analyses of suspected hybrid whales in Icelandic waters. (a) Mitochondrial DNA D‐loop Maximum Parsimony tree. Hybrids are highlighted with blue; (b) structure runs at 24 microsatellite loci: Red = fin whale, green = blue whale, red‐green = hybrids; (c) posterior probabilities of each hybrid to the different hybrids categories defined in NEWHYBRIDS. Only first generation (F1 hybrids) and backcross to fin whale are shown. Whale drawings were provided by Jón Baldur Hlíðberg©

All mitochondrial DNA sequences obtained from the six alleged hybrids (two between 1986 and 1989, three from 2013 to 2018 and the Skjálfandi Bay live individual) clustered with sequences of blue whale (Figure 3a), suggesting that their mothers were blue whales and their fathers fin whales. Nuclear genetic analyses using 24 microsatellite loci and Bayesian cluster analysis confirmed that five out of six hybrids sampled were first‐generation hybrids (Figure 3b), with assignment proportions equally split between parental species. The sixth whale, a hybrid male captured in 2018 (H2018‐2), exhibited higher assignment value (~70%) to fin whale. The posterior distribution analysis of hybrid individuals falling into different categories confirmed this finding and estimated a 92% posterior probability for this male to be a backcross (F2 or second‐generation hybrid) with fin whale (Figure 3c), while all other hybrids exhibited a 99% posterior probability of being first‐generation hybrids (Figure 3c). We concluded that the 2018 male hybrid was sired by a fin whale and mothered by a first‐generation hybrid female (see Table 1).

In addition, these findings highly support a unidirectional hybridization with male fin whales siring female blue whales (*Chi* ^2^ test, for F1 hybrids, *p* = .025 considering our data; *p* = .059 when all data presented in Table 1 were added).

## 4 DISCUSSION

Hybridization events among fin whale and blue whale have been reported since the 19th century but very little information exists on the directionality of such events and the reproductive status of hybrids. Here, we document a consistent pattern of male fin whales siring female blue whales, and the first occurrence of a second‐generation adult hybrid.

Until recently, hybridization among cetacean species has been thought to be a “dead‐end” because most hybrids were deemed to be infertile (Bérubé & Palsbøll, 2018). However, the observation of the 1986 hybrid fin × blue whale carrying a fetus in Icelandic waters and more recently of a hybrid between common minke (*B. acurostrata*) and Antarctic minke (*B. bonaerensis*) whales carrying a fetus resulting from a backcross mating with a male common minke whale in Norway (Glover et al., 2013) tend to support the idea that first‐generation hybrids might be, in certain circumstances, able to breed with one of the parental species. In the present study, the discovery of a second‐generation adult hybrid was surprising since only a pregnant hybrid female had been mentioned so far and proofs of living second‐generation adult marine mammals are crucially lacking. This discovery supports the fact that hybrids resulting from a successful mating of the two largest mammals on Earth can in some cases reproduce and that their offspring can survive to adulthood.

Fin and blue whales belong to the same genus, *Balaenoptera*, which diverged during the late Miocene between 10.5 and 7.5 Ma ago (Árnason, Lammers, Kumar, Nilsson, & Janke, 2018) with an estimate time to the most common ancestor of mysticetes in the late Oligocene (Sasaki et al., 2005). Whole‐genome sequencing studies investigating hybridization between blue whale and other rorquals confirmed the likely occurrence of ancestral introgression between fin and blue whales (Árnason et al., 2018; Westbury, Petersen, & Lorenzen, 2019); however, more recent, contemporary signatures are likely challenging to detect. Overall, it can be expected that fin x blue whale hybrids will exhibit reduced fitness, preventing backcross with both parental species (Árnason et al., 2018; Westbury et al., 2019). Yet, our discovery of the first second‐generation hybrid adult and the previous report of the 1986 pregnant hybrid female (Spilliaert et al., 1991) indicate that at least some hybrid fin × blue whales are fertile and can reproduce with both parental species, under certain environmental and demographic scenarios.

The identification of a second‐generation hybrid sired by a male fin whale and the observed directionality of hybridization (male fin whales siring female blue whales) might represent a concern for the future of the blue whale. A total of 7 out of the 8 hybrids genetically analyzed (see Table 1 for a summary) so far had a blue whale mother which suggests unidirectional hybridization (Árnason et al., 1991; Bérubé & Aguilar, 1998; Spilliaert et al., 1991). Unidirectional hybridization may occur for different reasons, such as size difference, ecological or behavioral bias, but one of the main potential explanations remains the “sexual selection hypothesis for unidirectional hybridization” (Wirtz, 1999). This hypothesis crucially depends on the abundance of the species involved in the hybridization event and suggests that the females of the rarer species, which initially reject allospecific males from the more common species, will eventually successfully mate with them due to the lack of conspecific males (Wirtz, 1999). Alternatively, the observed unidirectional hybridization could also be due to size constraints and the result of a purely physical/mechanical impossibilities for blue whale male to sire fin whale female. Today, abundance of the fin whales in the whole North Atlantic is estimated to be over 80,000 individuals (Aguilar & García‐Vernet, 2018; Pike, Gunnlaugsson, Mikkelsen, Halldórsson, & Víkingsson, 2019; IUCN, 2020) while blue whales abundance estimates vary between 2,100 and 4,000 (Sears & Perrin, 2018). Considering only the North Atlantic Central Region which includes Icelandic waters, the estimates are 36,800 (Pike et al., 2019) for the fin whale and 3,000 for the blue whale (Pike et al., 2019). The “sexual selection for unidirectional hybridization” will therefore likely result in the female of the rarest species, the blue whale, to be the maternal species of the hybrids, which coincides with our findings.

In the North Atlantic Ocean, most of the baleen whale species have now substantially recovered from historical whaling (IUCN, 2020), with few exceptions, such as the blue whale which has shown a slower recovery rate than most other whales (Thomas & Brownell, 2016). The inherent difficulty of blue whale to recover has been suggested to be due to resource competition (Reeves, Clapham, Brownell, & Silber, 1998) and climate change (Thomas & Brownell, 2016). Hybridization events among these large marine mammals are likely to be underestimated, and while population growth models considering hybridization events might have to be implemented to confirm this, we here raise awareness of an additional potential threat to blue whale population recovery. Blue whale is currently listed as endangered in the IUCN list and its global population remains at a very low level compared to prewhaling status although increase has been detected in the North Atlantic and Antarctic Oceans (Cooke, 2018; Pike et al., 2019; Sigurjónsson & Gunnlaugsson, 1990). A continued or increased hybridization and introgression with fin whale is likely to induce a loss of blue whale population reproductive output, thereby potentially affecting its recovery rate. At present, there are no frequency estimates of hybrids between fin and blue whales (the frequency of hybrids was 2% in our data in 2018, including the living hybrid). If hybridization is frequent and includes animals not visually identifiable from fin or blue whales in surveys, it might lead to the overestimation of population size of both species. This would be however more of concern for blue whale than fin whale, as its population size is about 25 times lower. It is therefore prudent to consider and monitor hybridization, where possible.

## COMPETING INTERESTS

“The authors declare no competing interests.”

## AUTHOR CONTRIBUTIONS

V.C. and S.D.H. conducted the genetic sampling protocol and registration of DNA tissues in the database. AKD, MRI, and MHR provided blue whale samples. C.P. and D.G. performed the analyses. C.P., B.Þ.E., D.G., G.Ó., S.S., G.A.V., and S.M. wrote the main paper. All authors discussed the results and implications and commented on the manuscript at all stages.

## Supporting information

Table S1

## Data Availability

The data used during the present study (information at 24 microsatellite loci and mtDNA control region) were deposited in the OSF open‐access database and are available at: https://osf.io/hfjgx/?view_only=deab3655a50243e0bcc5ce138bd05872
