# Supplementary material for: Evidence of unidirectional hybridization and second‐generation adult hybrid between the two largest animals on Earth, the fin and blue whales
Source: Evol Appl. 2020 Aug 28;14(2):314–21. doi: 10.1111/eva.13091 (PMC7896702; doi:10.1111/eva.13091)
Supplement: Supplementary file 1 — Table S1 [file EVA-14-314-s001.docx]

**Table S1.** Sample name, date of capture and sex of fin, hybrids and blue whales used in the present study. Fin whale sex was determined biologically while biopsies and blue whale sex were determined genetically.

| **SAMPLE CODE** | **Species** | **Date (d/m/y)** | **Sex** |
| --- | --- | --- | --- |
| F18-001 | Fin whale | 21.06.2018 | ♀ |
| F18-002 | Fin whale | 22.06.2018 | ♂ |
| F18-003 | Fin whale | 24.06.2018 | ♂ |
| F18-004 | Fin whale | 24.06.2019 | ♀ |
| F18-005 | Fin whale | 26.06.2018 | ♀ |
| F18-006 | Fin whale | 28.06.2018 | ♀ |
| F18-007 | Fin whale | 28.06.2018 | ♂ |
| F18-008 | Fin whale | 29.06.2018 | ♀ |
| F18-009 | Fin whale | 30.06.2018 | ♂ |
| F18-010 | Fin whale | 30.06.2018 | ♀ |
| F18-011 | Fin whale | 03.07.2018 | ♂ |
| F18-012 | Fin whale | 03.07.2018 | ♂ |
| F18-013 | Fin whale | 03.07.2018 | ♂ |
| F18-014 | Fin whale | 03.07.2018 | ♂ |
| F18-016 | Fin whale | 05.07.2018 | ♂ |
| F18-017 | Fin whale | 05.07.2018 | ♀ |
| F18-018 | Fin whale | 05.07.2018 | ♀ |
| F18-019 | Fin whale | 06.07.2018 | ♀ |
| F18-020 | Fin whale | 06.07.2018 | ♂ |
| F18-021 | Fin whale | 06.07.2018 | ♂ |
| F18-023 | Fin whale | 08.07.2018 | ♂ |
| F18-024 | Fin whale | 11.07.2018 | ♂ |
| F18-025 | Fin whale | 11.07.2018 | ♀ |
| F18-044 | Fin whale | 20.07.2018 | ♀ |
| F18-049 | Fin whale | 23.07.2018 | ♀ |
| F18-053 | Fin whale | 25.07.2018 | ♀ |
| F18-064 | Fin whale | 04.08.2018 | ♂ |
| F18-074 | Fin whale | 09.08.2018 | ♂ |
| F18-075 | Fin whale | 09.08.2018 | ♂ |
| F18-096 | Fin whale | 22.08.2018 | ♀ |
| F18-097 | Fin whale | 22.08.2018 | ♀ |
| F18-100 | Fin whale | 24.08.2018 | ♂ |
| F18-106 | Fin whale | 26.08.2018 | ♀ |
| F18-132 | Fin whale | 12.09.2018 | ♂ |
| H1986 | Hybrids | 18.06.1986 | ♀ |
| H1989 | Hybrids | 29.06.1989 | ♂ |
| HALIVE | Hybrids | 05.06.2014 | **♂** |
| H2013 | Hybrids | 21.6.2013 | ♀ |
| H2018-1 | Hybrids | 07.07.2018 | ♂ |
| H2018-2 | Hybrids | 23.08.2018 | ♂ |
| 2-99 | Blue whale | 29.06.1999 | ♂ |
| 3-99 | Blue whale | 30.06.1999 | ♀ |
| 4-99 | Blue whale | 01.07.1999 | ♂ |
| 6-99 | Blue whale | 1999 | ♂ |
| 7-99 | Blue whale | 1999 | ♀ |
| 8-99 | Blue whale | 1999 | ♂ |
| 9-99 | Blue whale | 1999 | ♀ |
| 10-99 | Blue whale | 1999 | ♂ |
| 11-99 | Blue whale | 1999 | ♂ |
| 12-99 | Blue whale | 1999 | ♂ |
| 13-99 | Blue whale | 1999 | ♂ |
| 14-99 | Blue whale | 1999 | NA |
| 15-99 | Blue whale | 1999 | ♀ |
| BM1-23-06-2011 | Blue whale | 23.06.2011 | ♂ |
| BM2-23-6-2011 | Blue whale | 23.06.2011 | ♀ |
| BM3-23-06-11 | Blue whale | 23.06.2011 | ♂ |
| BM6-25-6-11 | Blue whale | 25.06.2011 | ♀ |
| LK-BM01-2015 | Blue whale | 01.5.2016 | ♀ |
| 2-7-92-N2-SURTSEY | Blue whale | 02.07.1992 | ♀ |
| BM-07062015 | Blue whale | 07.05.2015 | ♂ |
| TAG60010 | Blue whale | **NA** | **NA** |
| BM1-08-06-2011 | Blue whale | 08.06.2011 | ♀ |
| BM-26-06-11-VOLCANO | Blue whale | 26.06.2011 | ♀ |
| S1011 | Blue whale | 25.08.2010 | ♂ |
| S1010 | Blue whale | 23.08.2016 | ♀ |
| EN5-2014-N2 | Blue whale | 29.05.2014 | ♂ |
| EN-3-2011-21-06-11 | Blue whale | 21.06.2011 | ♂ |
